# Survival of Fragmented BO_4_ Units in Highly Modified Rare-Earth-Rich Borate Glasses

**DOI:** 10.1021/acs.inorgchem.4c03264

**Published:** 2024-11-20

**Authors:** Shunta Sasaki, Atsunobu Masuno, Yutaka Yanaba, Hiroyuki Inoue, Takahiro Ohkubo

**Affiliations:** † Graduate School of Science and Technology, Hirosaki University, 3 Bunkyo-cho, Hirosaki, Aomori 036-8505, Japan; ‡ Graduate School of Engineering, Kyoto University, Kyotodaigaku-Katsura, Nishikyo-ku, Kyoto 615-8520, Japan; § Institute of Industrial Science, The University of Tokyo, 4-6-1 Komaba, Meguro-ku, Tokyo 153-8505, Japan; ∥ Graduate School of Engineering, Chiba University, 1-33 Yayoi-cho, Inage-ku, Chiba 263-8522, Japan

## Abstract

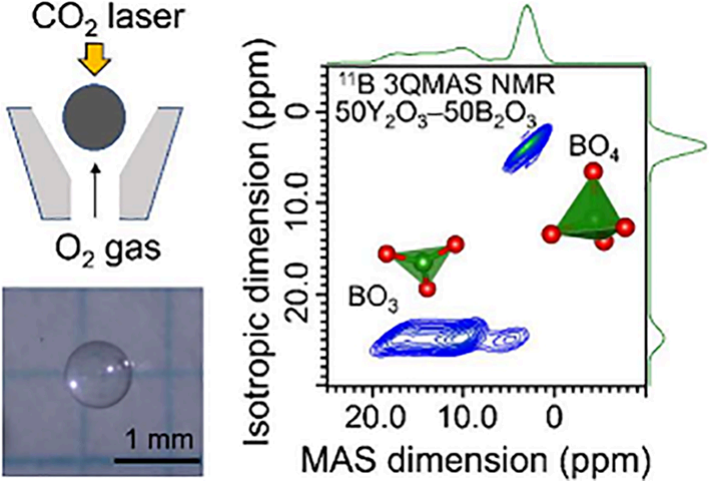

Highly modified La_2_O_3_–Y_2_O_3_–B_2_O_3_ ternary glasses were fabricated by using a levitation technique. The thermal and structural properties of (50 – *x*)La_2_O_3_–*x*Y_2_O_3_–50B_2_O_3_ glasses and (60 – *y*)La_2_O_3_–*y*Y_2_O_3_–40B_2_O_3_ glasses were investigated. Raman scattering spectra indicated that B atoms mainly formed isolated planar BO_3_ triangles similar to those of crystalline LaBO_3_. This process was independent of the ratio of La_2_O_3_ and Y_2_O_3_. ^11^B magic angle spinning nuclear magnetic resonance spectra confirmed that the BO_4_ units that should have disappeared in the glass with highly modified compositions remained as fragmented species. Approximately 4% of the B atoms formed BO_4_ in the 50La_2_O_3_–50B_2_O_3_ glass. This ratio increased with an increase in the Y_2_O_3_ content, and it reached its maximum value (15%) in the 50Y_2_O_3_–50B_2_O_3_ glass. Comparison of the electron density distribution was conducted using *ab initio* calculations of the LaBO_3_ and YBO_3_ crystals and indicated that more electrons localize near the atomic nuclei in the Y–O bond than in the La–O bonds. Comparison of the electron density distribution was conducted using *ab initio* calculations of the LaBO_3_ and YBO_3_ crystals and indicated that electrons in the Y–O bond localize near the atomic nuclei compared to those in the La–O bonds. Thus, the unconventional existence of BO_4_ in a highly modified glass is attributed to the increase in the level of Y^3+^, which causes the localization of electrons near the atomic nuclei. Thus, the ratio of BO_4_ and BO_3_ in highly modified glass can be controlled by tuning the glass content of modifier rare-earth oxides, which opens a new door to glass science.

## Abstract 2

La_2_O_3_−Y_2_O_3_−B_2_O_3_ ternary glasses were fabricated using a levitation technique. The local structure around the B atoms was investigated using several spectroscopy analyses. ^11^B triple-quantum magic angle spinning nuclear magnetic resonance spectra confirmed that the BO_4_ units that should have disappeared in the glass with highly modified compositions remained along with isolated BO_3_ units. These BO_4_ units were also fragmented, similar to the isolated BO_3_ units. Thus, La_2_O_3_- and Y_2_O_3_-rich borate glasses have a fragmented structure with no three-dimensional random network.

## Introduction

Along with SiO_2_ and P_2_O_5_, B_2_O_3_ is one of the most common network-forming oxides.^1,2^ In pure B_2_O_3_ glass, planar BO_3_ triangles connect at the corners, forming a boroxol ring network.^3,4^ The addition of modifier oxides, such as alkali oxides (A_2_O, where A is an alkali metal element) and alkaline-earth oxides (MO, where M is an alkaline-earth metal element), to B_2_O_3_ results in the formation of BØ_4_^–^ without generating nonbridging oxygens, which cause complex changes in the structural groups in borate glass.^5,6^*N*_4_ is the fraction of the BO_4_ units to all of the BO_*n*_ units (*n* = 3 or 4). *N*_4_ increases with modifier oxide content and reaches its maximum at ∼40 mol %. A further increase in the modifier oxide content in the borate glass breaks the B–O–B network and generates nonbridging oxygens.^6−8^ The complex change in the local structure around the B atoms causes the borate anomaly, i.e., various physical properties, such as the coefficient of thermal expansion, density, and thermal conductivity of the material, to change nonlinearly with the addition of modifier oxides.^9,10^ Therefore, the composition–structure–property relationship in the local structure around the B atoms in borate glasses has been attracting a considerable amount of attention.^11−15^

**Figure 1:**
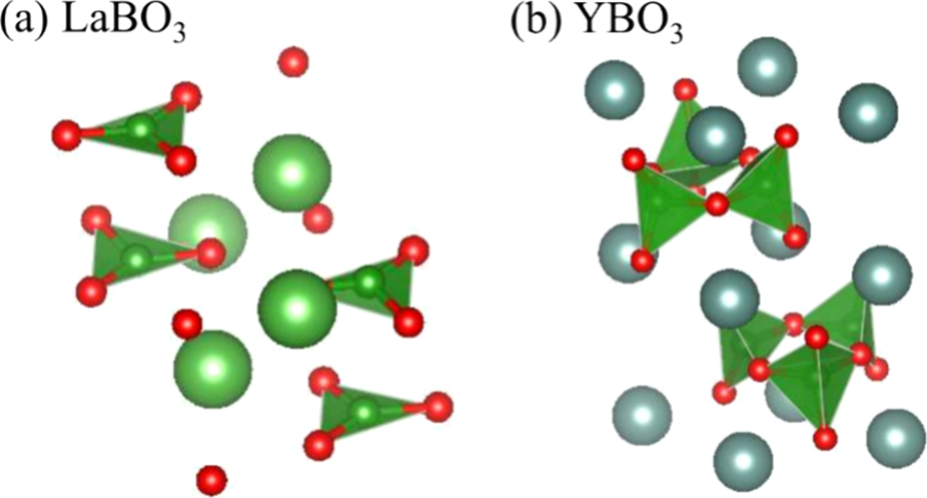
Local structures of (a) crystalline LaBO_3_ and (b) YBO_3_ drawn using VESTA.^20^

Few reports have investigated the structure and physical properties of highly modified borate glasses because, with an increasing modifier oxide content in the material, the network breaks, hindering glass formation. However, the number of recent research studies on highly modified borate glass has been increasing. Chryssikos et al. investigated the Raman scattering spectra of alkali borate glasses with a composition range of 2 ≤ O/B ≤ 3.^16^ When borate glasses with different alkali cations and an O/B ratio of 2.67 were compared, it was found that lower-ionic field Cs and Rb cations did not appear to promote the formation of pyroborate B_2_O_5_^4–^ and isolated orthoborate BO_3_^3–^ triangular units. Instead, they favor the formation of tetrahedral borate arrangements with the orthoborate composition, i.e., BØ_2_O_2_^3–^ tetrahedra with two bridging (Ø) and two nonbridging (O) oxygen atoms. In contrast, glasses containing higher-ionic field cations were dominated by B_2_O_5_^4–^ and BO_3_^3–^ species with boron in 3-fold coordination. In contrast, glasses containing higher-ionic field cations were dominated by them. Winterstein-Beckmann et al. investigated the thermal and vibrational properties of supermodified borate glasses, i.e., MnO–SrO–B_2_O_3_^17^ and Eu_2_O_3_–SrO–B_2_O_3_ glasses.^18^ Raman scattering and infrared absorption spectra indicated that BO_3_^3–^ and B_3_O_9_^9–^ formed at highly modified compositions (O/B ≥ 3). BO_3_^3–^, which can be found in crystalline LaBO_3_, is an isolated planar BO_3_ triangle with three nonbridging oxygens, whereas B_3_O_9_^9–^, which is found in crystalline YBO_3_, is formed by connecting three BØ_2_O_2_^3–^ ions at two corners of each unit (Figure 1). Pyroborate B_2_O_5_^4–^ and isolated BO_3_^3–^ are the main components of 50MnO–25SrO–25B_2_O_3_ glass (O/B = 3) with a small amount of B_3_O_9_^9–^, which was inferred from a weak absorption peak in the infrared spectrum. BO_4_ vibrational modes are also apparent in Raman spectra, even though the stretching of B–O overlaps with other units, ring breathing modes, and more are apparent, though infrared has no overlap and is much more unambiguous. In 60MnO–20SrO–20B_2_O_3_ glass, which involves a higher degree of modification (O/B = 3.5), the amount of B_3_O_9_^9–^ and BO_3_^3–^ increased and B_2_O_5_^4–^ remained. The fragmentation of borate units is attributed to the decrease in the glass transition temperature (*T*_g_), which increases with the modifier oxide content. On the contrary, the increase in *T*_g_ for the highly modified 60MnO–20SrO–20B_2_O_3_ glass was connected to increased network connectivity with formation of B_3_O_9_^9–^. This is considered a “second” borate anomaly.^17−19^

Herein, La_2_O_3_–B_2_O_3_ binary glasses with high La_2_O_3_ contents (≥30 mol %), which could not be synthesized via a melt–quench method, were synthesized using a levitation technique.^21^ The La_2_O_3_-rich borate glasses exhibited high thermal stability, a high refractive index, low wavelength dispersion, and additional infrared transparency. ^11^B magic angle spinning (MAS) nuclear magnetic resonance (NMR) spectra and vibrational analysis indicated that all of the B atoms formed isolated BO_3_^3–^ units without any B–O–B networks or BO_4_ units in the samples with La_2_O_3_-rich compositions (O/B ≥ 3). Structural analysis of the Gd_2_O_3_–B_2_O_3_ binary glasses indicated that the dominant structural units of isolated BO_3_ remained unchanged in the range of 3 ≤ O/B ≤ 3.75.^22^ Further comprehensive study of the 50R_2_O_3_–50B_2_O_3_ binary systems (R is a rare-earth element) indicated that isolated BO_3_ units formed in abundance in 50R_2_O_3_–50B_2_O_3_ glasses, irrespective of the kinds of R.^23^ High-energy X-ray diffraction (XRD) revealed that the atomic arrangement around the B and R atoms of the 50R_2_O_3_–50B_2_O_3_ glasses resembled that of crystalline *v*-RBO_3_.^24^ The unconventional glass formation in the samples with highly modified compositions was attributed to disorder in the atomic arrangement caused by a slight displacement from the ordered crystalline structure.

Recently, Topper et al. reported that in Li_2_O–R_2_O_3_–B_2_O_3_ ternary glasses, isolated BO_3_ was a dominant structural unit and no structural change was observed for O/B = 3 compositions.^25^

In this study, comprehensive structural investigations around the B atoms in highly modified rare-earth-rich La_2_O_3_–Y_2_O_3_–B_2_O_3_ glasses were conducted using Raman scattering spectroscopy, ^11^B MAS NMR spectroscopy, and ^11^B triple-quantum (3Q) MAS NMR spectroscopy.^26^ Crystalline (*c*-) LaBO_3_ (space group *Pmcn*) and YBO_3_ (space group *Cmcm*) were also investigated as references.^27,28^

## Experimental Procedures

The highly modified region of a La_2_O_3_–Y_2_O_3_–B_2_O_3_ ternary system synthesized by using a levitation technique was examined. La_2_O_3_, Y_2_O_3_, and H_3_BO_3_ powders were mixed and pressed into pellets at stoichiometric compositions. The pellets were annealed at 600 °C for 12 h in an air atmosphere to decompose H_3_BO_3_ into B_2_O_3_. The pellets were then ground and pelletized. Next, the pellets were sintered at 1000 °C for 24 h in an air atmosphere and then crushed to pieces to be suitable targets for an aerodynamic levitation (ADL) furnace. These pieces were placed on the nozzle of the ADL furnace and levitated by using an O_2_ gas flow. A CO_2_ laser was applied to melt the levitated sample. The laser power was turned off to rapidly cool the melt to room temperature and allow it to solidify. A spherical sample (diameter of ≈1–3 mm) was obtained. Crystalline LaBO_3_ and YBO_3_ were prepared through a conventional solid-state reaction to act as reference materials. Their formation was confirmed by using Cu Kα XRD (Rigaku SmartLab) measurements (Figure 1). Glass formation was also confirmed through XRD measurements.

The *T*_g_ and crystallization temperature (*T*_X_) of the glasses obtained were determined using thermogravimetric–differential thermal analyses (NETZSCH STA 2500 Regulus) from room temperature to 1000 °C at a heating rate of 10 °C/min in an air atmosphere. Before the physical properties were measured, the glasses were annealed near *T*_g_ for 10 min to remove any internal stresses. The density, ρ, of the glasses was measured by using a gas pycnometer (Micromeritics AccupycII 1340). The packing density, *D*_p_, was calculated using eq 1:
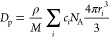
1where *M* is the molecular weight of the glass, *c*_*i*_ is the molar concentration of the *i*th ion, *N*_A_ is Avogadro’s number, and *r*_*i*_ is the ionic radius of the *i*th ion. The ionic radii of eight-coordinated La^3+^ (*r*_La_ = 1.16 Å), eight-coordinated Y^3+^ (*r*_Y_ = 1.02 Å), three-coordinated B^3+^ (*r*_BIII_ = 0.01 Å), four-coordinated B^3+^ (*r*_BIV_ = 0.11 Å), and two-coordinated O^2–^ (*r*_O_ = 1.35 Å) were adopted in the calculations.^29^*N*_4_ was estimated by using ^11^B MAS NMR.

Raman scattering spectra were obtained using a JASCO NRS-4500 laser Raman spectrometer employing a 532 nm laser light as the incident light source. ^11^B NMR spectroscopy measurements were performed on a JEOL JNM-ECA500 spectrometer at 11.74 T (^1^H, 500 MHz) equipped with an MAS probe head (spinning rate of 15 kHz) and a zirconia rotor (diameter of 4 mm). The NMR spectra were recorded at μ/6 pulses (1.0 μs) and a relaxation delay of 30 s with 64 accumulated signal transients. ^11^B chemical shifts were expressed in parts per million relative to a 1 M boric acid aqueous solution at 19.5 ppm. The NMR spectra were decomposed using “dmfit”.^30^^11^B 3QMAS NMR spectra were recorded using three pulses: a triple-quantum excitation pulse (4.8 μs), a single-quantum conversion pulse (1.7 μs), and a Z filter selective excitation pulse at a relaxation delay of 5 s with 24 accumulated signal transients.

The trivalent bonding states with the BO_*n*_ unit in *c*-LaBO_3_ and *c*-YBO_3_ were analyzed on the basis of the electron density obtained using density functional theory (DFT) calculations. DFT calculations were performed using the Vienna *ab initio* calculation package^31^ based on the projector-augmented-wave method.^32^ The electron exchange correlation energy was described using the generalized gradient approximation with the Perdew–Burke–Ernzerhof (GGA–PBE) revised functional for solids.^33,34^ The structures in Figure 1a,b were used as the initial structures of *c*-LaBO_3_ and *c*-YBO_3_, respectively, in the DFT calculations.^27,28^ Atomic positions and cell dimensions were optimized by using an ionic relaxation routine. The convergence criteria for the self-consistent field cycle and ionic relaxation were 10^–8^ eV and 10^–3^ eV/Å, respectively, for the maximum force on each atom. The cutoff energy for the plane-wave basis was set to 400 eV as required by the pseudopotentials. The *k*-point sampling for LaBO_3_ and YBO_3_ was distributed within the Brillouin zone on 6 × 5 × 5 and 3 × 5 × 3 Monkhorst–Pack grids,^35^ respectively.

## Results and Discussion

### Glass-Forming Region

**Figure 2:**
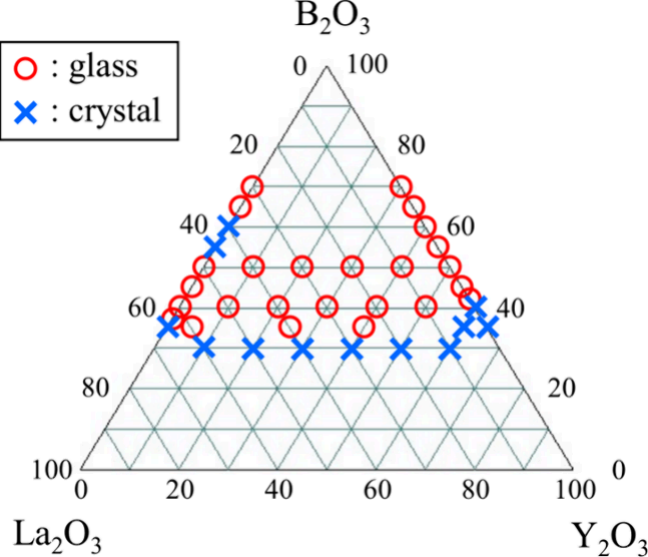
Glass-forming region of the La_2_O_3_–Y_2_O_3_–B_2_O_3_ ternary system. Circles and crosses represent glass formation and crystallization, respectively.

Figure 2 shows the glass-forming region of the La_2_O_3_–Y_2_O_3_–B_2_O_3_ ternary system. The upper content limits of the rare-earth oxides (La_2_O_3_ and Y_2_O_3_) in the La_2_O_3_–B_2_O_3_ and Y_2_O_3_–B_2_O_3_ binary systems were 63 and 58 mol %, respectively.^23^ In the ternary system, colorless and transparent glasses were obtained when the total contents of La_2_O_3_ and Y_2_O_3_ were 60 and 50 mol %, respectively, except in the composition of 60Y_2_O_3_–40B_2_O_3._ In addition, when the total amount of La_2_O_3_ and Y_2_O_3_ was 65 mol %, glasses were obtained in the La_2_O_3_-rich region (≥40 mol % La_2_O_3_). In contrast, glass was not obtained in the region where the total amount of La_2_O_3_ and Y_2_O_3_ was 70 mol %.

### Physical Properties

The composition dependences of *T*_g_ and *T*_X_ of the (50 – *x*)La_2_O_3_–*x*Y_2_O_3_–50B_2_O_3_ glasses (50LY glasses) and (60 – *y*)La_2_O_3_–*y*Y_2_O_3_–40B_2_O_3_ glasses (60LY glasses) are summarized in Table 1. The *T*_g_ of the 50LY glasses was almost constant at ∼750 °C in the *x* range of 0–30; however, it increased to 792 °C when *x* exceeded 30. The *T*_g_ of the 60LY glasses gradually increased from 733 to 771 °C in the *y* range of 0–50. The *T*_X_ of both the 50LY and 60LY glasses varied around 820 °C and exhibited little composition dependence. The difference between *T*_g_ and *T*_X_ (Δ*T* = *T*_X_ – *T*_g_) is a measure of the stability of the glass against crystallization. Similar to *T*_X_, hardly any composition dependence of *ΔT* was observed. *ΔT* was <100 °C irrespective of the compositional ratio of La_2_O_3_ and Y_2_O_3_, indicating the relatively low glass stability.

**Table 1:** Glass Transition (*T*_g_, degrees Celsius) and Crystallization (*T*_X_, degrees Celsius) Temperatures, *ΔT* (=*T*_X_ – *T*_g_), Densities (ρ, grams per cubic centimeter), Packing Densities (*D*_p_), and *N*_4_ Values of the (50 – *x*)La_2_O_3_–*x*Y_2_O_3_–50B_2_O_3_ and (60 – *y*)La_2_O_3_–*y*Y_2_O_3_–40B_2_O_3_ Glasses

	*T*_g_	*T*_*x*_	Δ*T*	ρ	*D*_p_	*N*_4_
50La_2_O_3_–50B_2_O_3_	752.0	811.4	59.4	5.54 ± 0.01	0.632	0.04
40La_2_O_3_–10Y_2_O_3_–50B_2_O_3_	749.0	774.0	25.0	5.49 ± 0.02	0.653	0.06
30La_2_O_3_–20Y_2_O_3_–50B_2_O_3_	756.2	845.1	88.9	5.35 ± 0.01	0.664	0.09
20La_2_O_3_–30Y_2_O_3_–50B_2_O_3_	752.2	819.1	66.9	4.88 ± 0.01	0.634	0.11
10La_2_O_3_–40Y_2_O_3_–50B_2_O_3_	769.5	844.2	74.7	4.67 ± 0.01	0.638	0.13
50Y_2_O_3_–50B_2_O_3_	792.2	835.0	42.8	4.55 ± 0.01	0.655	0.15
60La_2_O_3_–40B_2_O_3_	733.0	810.8	77.8	5.53 ± 0.01	0.578	0.04
50La_2_O_3_–10Y_2_O_3_–40B_2_O_3_	750.0	839.0	89.0	5.39 ± 0.01	0.583	0.06
40La_2_O_3_–20Y_2_O_3_–40B_2_O_3_	751.1	836.0	84.9	5.22 ± 0.01	0.586	0.08
30La_2_O_3_–30Y_2_O_3_–40B_2_O_3_	762.0	799.0	37.0	5.14 ± 0.01	0.601	0.10
20La_2_O_3_–40Y_2_O_3_–40B_2_O_3_	769.0	815.0	46.0	4.98 ± 0.01	0.606	0.13
10La_2_O_3_–50Y_2_O_3_–40B_2_O_3_	771.0	826.0	55.0	4.89 ± 0.02	0.623	0.15

The values of ρ and *D*_p_ of the 50LY and 60LY glasses are listed in Table 1. The ρ values of both glass compositions decreased linearly with an increase in Y_2_O_3_ content because the atomic weight of Y is smaller than that of La. The *D*_p_ of the 50LY glasses increased from 0.632 to 0.664 at in the *x* range of 0–20 with an increase in Y_2_O_3_ content, and it then decreased to 0.634 at *x* = 30 and increased again to 0.655 in the *x* range of 30–50. The *D*_p_ of the 60LY glasses increased linearly from 0.578 to 0.623 with Y_2_O_3_ content. The physical properties of La_2_O_3_–B_2_O_3_ and Y_2_O_3_–B_2_O_3_ have been evaluated in previous studies.^21,23^

### Raman Scattering Spectroscopy

**Figure 3:**
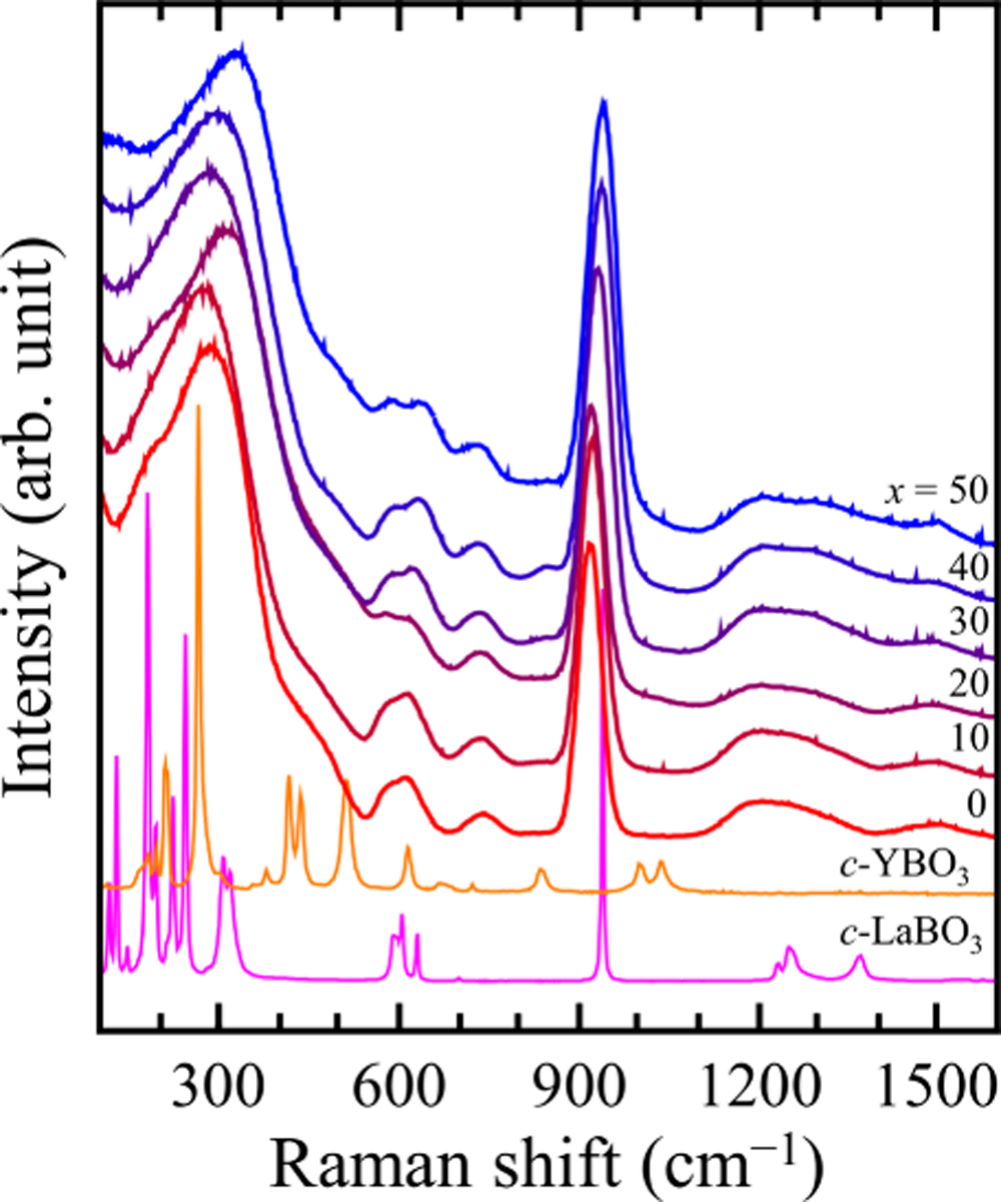
Raman scattering spectra of the (50 – *x*)La_2_O_3_–*x*Y_2_O_3_–50B_2_O_3_ glasses and crystalline YBO_3_ and LaBO_3_.

The Raman scattering spectra of the 50LY glasses and crystalline LaBO_3_ and YBO_3_ are shown in Figure 3. The spectral shapes of the 50LY glasses were almost identical, indicating that the small changes in the local environment due to substituting La with Y do not change significantly the overall network structure of the 50LY glasses. Band assignment was performed using the same method as in previous studies of R_2_O_3_–B_2_O_3_ binary glasses.^22,23^ The band at 320 cm^–1^ was assigned to the R–O (R = La and/or Y) vibration. Four fundamental modes of B–O vibration of the BO_3_^3–^ group, i.e., *ν*_1_ for the symmetric stretching mode, *ν*_2_ for the out-of-plane bending mode, *ν*_3_ for the asymmetric stretching mode, and *ν*_4_ for the in-plane bending mode, were observed at ∼950, ∼750, ∼1300, and ∼600 cm^–1^, respectively. These bands correspond to those observed in crystalline LaBO_3_, although the intensity of the 750 cm^–1^ band is considerably smaller. The bands attributed to the B–O–B stretching vibrations of metaborates (observed at 660 and 700 cm^–1^ for the ring and chain, respectively) and pyroborate (850 cm^–1^), which are observed in B_2_O_3_-rich compositions,^14,16,21,36^ were not confirmed in the 50LY glasses or in *c*-LaBO_3_. Furthermore, hardly any similarity was observed between the spectral shapes of the glasses and *c*-YBO_3_ except for that of the R–O vibration band. The spectrum of *c*-YBO_3_ did not show the vibration bands associated with the BO_3_^3–^ group. The bands observed were assigned to the vibration of the B_3_O_9_^9–^ group formed by three BØ_2_O_2_^3–^ units. The B–O vibrations of the B_3_O_9_^9–^ group, i.e., B–O^–^ terminal stretching at 1000 cm^–1^, B–O ring stretching at 830 cm^–1^, B–O ring bending at 410 and 510 cm^–1^, and B–O^–^ terminal bending at 260 cm^–1^, were observed.^18,24^ As a slight composition dependence, very weak peaks at ∼840 cm^–1^ were observed at *x* values of 30, 40, and 50. The peak in this region may be attributed to pyroborate B_2_O_5_^4–^ observed in B_2_O_3_-rich Y_2_O_3_–B_2_O_3_ in Figure S1, B_3_O_9_^9–^ observed in the *c*-YBO_3_ and 60MnO–20SrO–20B_2_O_3_ glass, or both.^17^ However, it is not possible to make accurate assignments. These results indicate that the local environment around the B atoms in the glass mainly contains isolated BO_3_ units with no B–O–B networks.

### ^11^B NMR Spectroscopy

**Figure 4:**
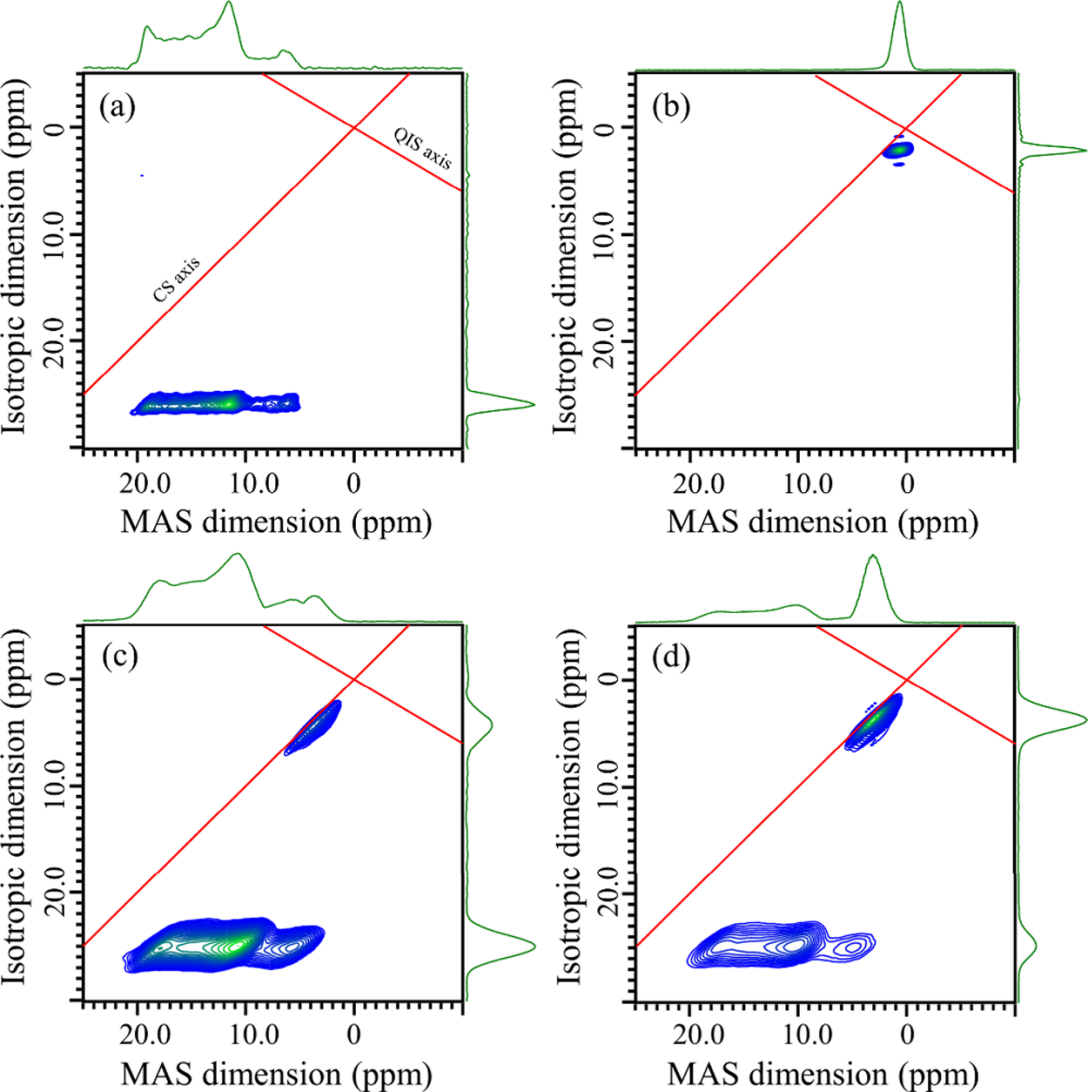
^11^B 3QMAS NMR spectra of (a) *c*-LaBO_3_, (b) *c*-YBO_3_, (c) 50La_2_O_3_–50B_2_O_3_ glass, and (d) 50Y_2_O_3_–50B_2_O_3_ glass. A chemical shift (CS) axis with a slope of 1 and a quadrupolar induced shift (QIS) axis with a slope of −10/17 are inserted.

Figure 4a–d show the ^11^B 3QMAS NMR spectra of *c*-LaBO_3_, *c*-YBO_3_, 50La_2_O_3_–50B_2_O_3_ glass, and 50Y_2_O_3_–50B_2_O_3_ glass, respectively. The ^11^B 3QMAS NMR spectra of the (50 – *x*)La_2_O_3_–*x*Y_2_O_3_–50B_2_O_3_ and (60 – *y*)La_2_O_3_–*y*Y_2_O_3_–40B_2_O_3_ ternary glasses are shown in Figures S2 and S3, respectively.

As shown in Figure 1a,b, all of the B atoms in *c*-LaBO_3_ form isolated triangle planar BO_3_ units;^26^ on the contrary, those in *c*-YBO_3_ form BØ_2_O_2_^3–^ units and the three units each connect through corner sharing to build a B_3_O_9_^9–^ ring structure.^28^ The spectral peaks of both crystals can be easily assigned to the local environment around the B atoms. A single broad peak parallel to the MAS axis of *c*-LaBO_3_ was associated with three-coordinated B atoms undergoing nuclear quadrupolar interaction, and hence, it was assigned to an isolated BO_3_ unit. A sharp peak was observed in the spectrum of *c*-YBO_3_, which is due to the BØ_2_O_2_^3–^ with two bridging oxygens (O_b_) and two nonbridging oxygens (*O*_nb_) in the B_3_O_9_^9–^ ring. The peak observed in *c*-YBO_3_ was not detected in the *c*-LaBO_3_ spectrum, and vice versa.

The spectra of the 50La_2_O_3_–50B_2_O_3_ glass and the 50Y_2_O_3_–50B_2_O_3_ glass exhibited similar shapes. Two main peaks were clearly observed in both glass compositions, which were not observed in the spectra of the crystalline phases. In the spectra of *c*-LaBO_3_ and *c*-YBO_3_, the peak at a high-parts per million shift can be attributed to isolated BO_3_ units, and the peak at a low-parts per million shift originates from the BO_4_ units. The 3QMAS NMR spectra confirmed, on the basis of the high- and low-parts per million peaks, that BO_3_ and BO_4_ were present in the glasses, even though it was difficult to find a trace of the BO_4_ units in a previous report on La_2_O_3_-rich borate glasses using ^11^B MAS NMR measurements and Raman scattering spectra. BO_4_ units were detected in the highly modified rare-earth-rich borate glasses. This result is supported by the observation of a few traces of BO_4_ obtained from NMR measurements of 55La_2_O_3_–45B_2_O_3_ applying a high field (19.6 T) by Swanson et al.^37^

**Figure 5:**
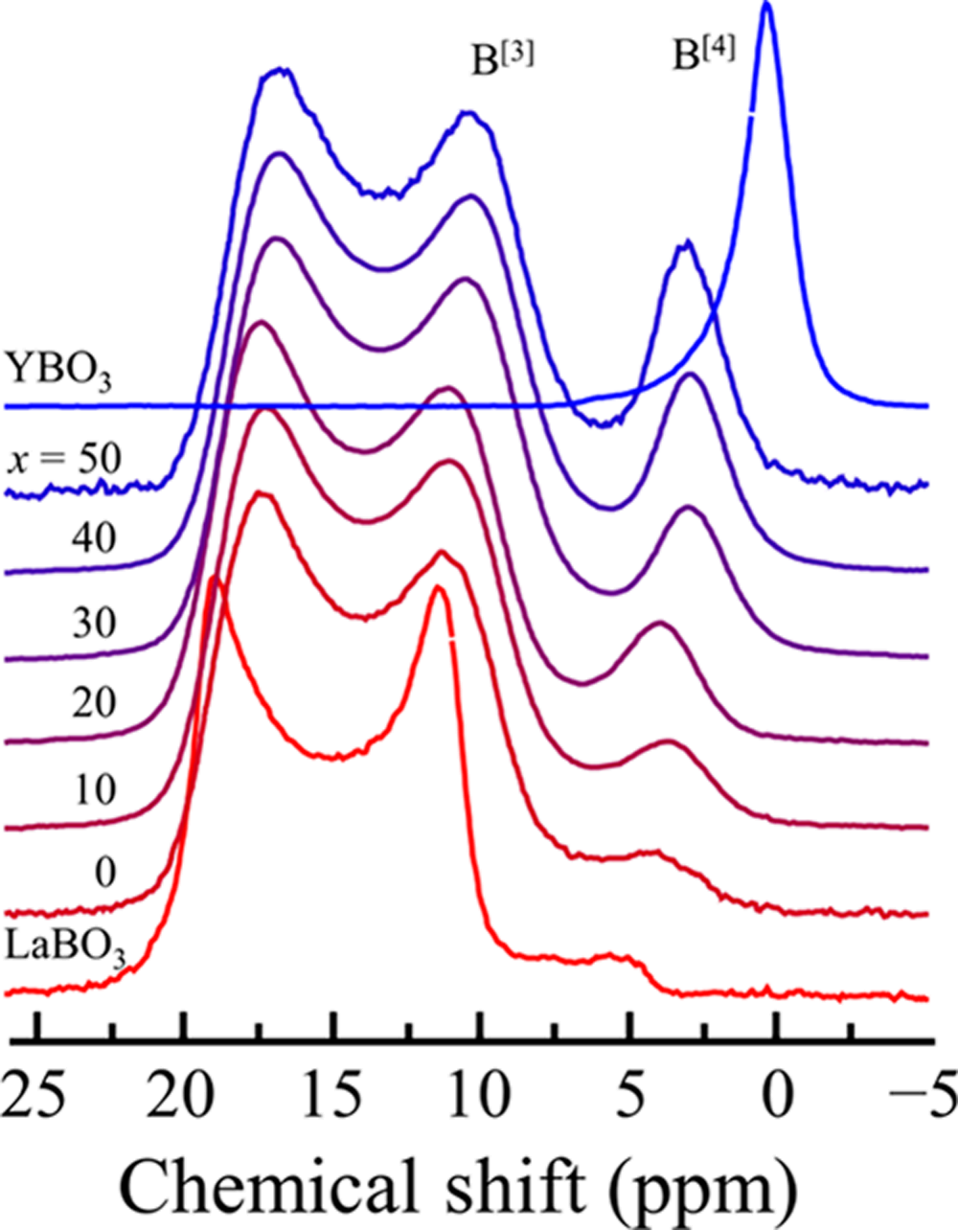
^11^B NMR spectra of the (50 – *x*)La_2_O_3_–*x*Y_2_O_3_–50B_2_O_3_ ternary glasses, *c*-LaBO_3_, and *c*-YBO_3_.

The ^11^B MAS NMR spectra of the 50LY glasses, *c*-LaBO_3_, and *c*-YBO_3_ are shown in Figure 5. In the spectrum of *c*-LaBO_3_, the peak associated with the isolated BO_3_ unit exhibited a broad shape at 3–22 ppm. In the *c*-YBO_3_ spectrum, the asymmetrical sharp peak associated with the BØ_2_O_2_^3–^ units in the B_3_O_9_^9–^ ring was located at ∼0 ppm. The spectrum exhibits a mild slope in the higher-parts per million direction.

Similar to the case of *c*-LaBO_3_, in which isolated BO_3_ peaks were observed, BO_3_ peaks were clearly observed in the spectra of the 50LY glasses.^21,38^ No considerable difference was observed between the peak positions and shapes of *c*-LaBO_3_ and the glasses. It was difficult to identify the BO_4_ peak in the spectrum of the 50La_2_O_3_–50B_2_O_3_ glass, which was the case in our previous study.^21^ However, the sharp peak of BØ_2_O_2_^3–^ in the 3QMAS NMR spectrum of the 50Y_2_O_3_–50B_2_O_3_ glass was clearly observed with a relatively strong intensity. The peak position clearly shifted to a higher-parts per million side compared with that of *c*-YBO_3_. This means that the BO_4_ units in the glass are different from those in the B_3_O_9_ ring structure of *c*-YBO_3_. The ^11^B MAS NMR spectrum of *z*Y_2_O_3_–(100 – *z*)B_2_O_3_ binary glasses, shown in Figure S4, reveals that the peak assigned to the BO_4_ unit shifts to a higher parts per million as *z* increases from 30 to 40, but no change is observed at *z* values of ≥40. The BO_4_ peak is expected to shift, reflecting the structure. However, the presence of peaks at similar parts per million at *z* values of ≥40 indicates that the presence of Y^3+^, which increases with Y_2_O_3_ content, does not contribute to the shift. From these facts, it is inferred that the structural units composed of BO_4_ are also different between the B_2_O_3_-rich and Y_2_O_3_-rich compositions. Considering that all BO_3_ units in (50 – *x*)La_2_O_3_–*x*Y_2_O_3_–50B_2_O_3_ ternary glasses are isolated BO_3_ without bridging oxygen, BO_4_ may also have a smaller number of bridging oxygens, such as in isolated BO_4_^5–^. The relative intensity of the BO_4_ peak in the ^11^B MASNMR spectra of (50 – *x*)La_2_O_3_–*x*Y_2_O_3_–50B_2_O_3_ ternary glasses decreased with the Y_2_O_3_ content, indicating a decrease in the ratio of BO_4_. Furthermore, the BO_4_ peak shifted to a higher-parts per million side with a decrease in Y_2_O_3_ content, suggesting that the increase in La_2_O_3_ content may increasingly break the connectivity of BO_4_.

### Estimation of NMR Parameters

The NMR spectrum is affected by a second-order quadrupolar interaction that depends on three parameters: (1) isotropic chemical shift (δ_iso_), which reflects the degree of connectivity of the boron atoms, (2) quadrupole coupling constant (*C*_Q_), which is a measure of the symmetry for a coordination shell around a nucleus, and (3) quadrupole asymmetry parameter (η), which describes the deviation of the electric field gradient from the axial symmetry.^39^ For borate groups, δ_iso_ is sensitive to the number of B–O coordinations, the nearest neighbor of the boron. Furthermore, especially in BO_3_, the number of O_b_ atoms associated with the second coordination sphere has been found to affect δ_iso_.^37^ On the contrary, the effect of the number of O_b_ atoms in BO_4_ is not detailed. *C*_Q_ is a function of the electric field gradient at the nucleus, *eq*, and the nuclear quadrupole moment, *eQ*, which can be accordingly defined using eq 2:
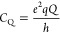
2where *h* is Planck’s constant. These three parameters of the glasses were estimated from the ^11^B MAS NMR spectral decompositions (the details are illustrated in Figure S5). The ^11^B MAS NMR spectral decomposition of the BO_4_ units was performed using a Gaussian/Lorentzian curve, whereas that of the BO_3_ units was performed using quadrupolar lines, including a Gaussian distribution.^40^ The relationship between different *O*_nb_ or structural units and δ_iso_ and *C*_Q_ in BO_4_ is uncertain, as it has not been reported. However, on the basis of previous studies of BO_3_,^38^ it is expected that these should be individually assigned according to the *O*_nb_ and/or structural unit. The BO_3_ and BO_4_ peaks are distinct, but further detailed peak differences are not clearly observed, making it difficult to assign them to each structural unit. Therefore, in this study, fitting was attempted for both BO_3_ and BO_4_ into a maximum of two components, at higher and lower parts per million, respectively. However, it should be noted that even this fitting may not be sufficient. In the (50 – *x*)La_2_O_3_–*x*Y_2_O_3_–50B_2_O_3_ (Figure S5 and Table 2) and (60 – *y*)La_2_O_3_–*y*Y_2_O_3_–50B_2_O_3_ ternary (Figure S6 and Table S1) glasses, NMR parameters for both BO_3_ and BO_4_ were determined as one component for all compositions. In the *z*Y_2_O_3_–(100 – *z*)B_2_O_3_ binary glasses (Figure S7 and Table S2), the parameters for *z* values of ≥50 were evaluated like those of the ternary glasses. For *z* values of ≤45, the parameters were determined by taking into account additional components of lower parts per million. The δ_iso_ values in both the BO_3_ and BO_4_ peaks of the (50 – *x*)La_2_O_3_–*x*Y_2_O_3_–50B_2_O_3_ ternary glasses with Y_2_O_3_-rich compositions shifted toward a lower parts per million. It should be noted that there is a margin of error of a few percent in determining NMR parameters. The *C*_Q_ tended to increase slightly with Y_2_O_3_ content. These trends were similar for the (60 – *y*)La_2_O_3_–*y*Y_2_O_3_–50B_2_O_3_ ternary (Table S1) glasses.

**Table 2:** ^11^B NMR Parameters, Including Isotropic Chemical Shifts (δ_iso_), Quadrupole Coupling Constants (*C*_Q_), and Quadrupole Asymmetry Parameters (η) and Gaussian Broadening of the (50 – *x*)La_2_O_3_–*x*Y_2_O_3_–50B_2_O_3_ Ternary Glasses Extracted from Spectral Decompositions Using the Gaussian/Lorentzian and Q MAS 1/2 Models for the BO_4_ and BO_3_ Peaks, Respectively, and ^11^B 3QMAS NMR Parameters, Including Chemical Shifts (*δ*_cs_) and Magnitudes of the Nuclear Quadrupolar Interaction (*P*_Q_) of the BO_4_ and BO_3_ Peaks

	δ_iso_ (ppm)	*C*_Q_ (MHz)	η	Gaussian broadening (Hz)	δ_cs_ (ppm)	*P*_Q_ (MHz)		δ_iso_ (ppm)	*C*_Q_ (MHz)	η	Gaussian broadening (Hz)	δ_cs_ (ppm)	*P*_Q_ (MHz)
50La_2_O_3_–50B_2_O_3_							40La_2_O_3_–10Y_2_O–50B_2_O_3_						
BO_4_	3.8	–	–	–	4.1	0.76	BO_4_	3.4	–	–	–	4.0	0.82
BO_3_	20.9	2.66	0.08	458.3	20.4	2.84	BO_3_	21.0	2.70	0.17	448.7	20.4	2.84
50Y_2_O_3_–50B_2_O_3_							30La_2_O_3_–20Y_2_O–50B_2_O_3_						
BO_4_	2.9	–	–	–	3.6	0.77	BO_4_	3.8	–	–	–	5.0	1.18
BO_3_	20.6	2.72	0.18	409.9	20.3	2.83	BO_3_	21.2	2.70	0.16	424.8	21.4	3.04
*c*-LaBO_3_							20La_2_O_3_–30Y_2_O–50B_2_O_3_						
BO_4_	–	–	–	–			BO_4_	2.8	–	–	–	3.7	0.86
BO_3_	21.9	2.67	0.06		21.2	2.87	BO_3_	20.6	2.71	0.20	436.0	19.9	2.88
*c*-YBO_3_							10La_2_O_3_–40Y_2_O–50B_2_O_3_						
BO_4_ I	1.2	–	–	–	1.6	0.96	BO_4_	2.8	–	–	–	3.5	0.82
BO_4_ II	0.3	–	–	–			BO_3_	20.6	2.73	0.20	416.8	19.8	2.90

The parameter *P*_Q_, which expresses the magnitude of the nuclear quadrupole interaction, correlates with *C*_Q_ and η as shown in eq 3
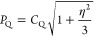
3

*P*_Q_ can be estimated using eq 4 on the basis of the 3QMAS NMR spectra
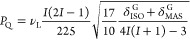
4where δ_MAS_^G^ and δ_ISO_^G^ are the centers of gravity of the peaks on the MAS axis and on the isotropic axis, respectively, *ν*_L_ is the Larmor frequency, *I* is the nuclear spin (^3^/_2_), and η is the asymmetry parameter. The intrinsic chemical shift, δ_cs_, in which the effect of a second-order quadrupolar interaction is suppressed, can also be estimated by using eq 5
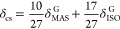
5

The values of *P*_Q_ and δ_cs_ obtained from 3QMAS NMR are listed in Table 2. Considering the differences in measurement methods, it was evaluated that the *P*_Q_ values obtained from ^11^B 3Q MAS NMR are consistent with the *C*_Q_ and η values obtained from ^11^B MAS NMR.

The *P*_Q_ values for both BO_3_ and BO_4_ showed comparable values for the 50La_2_O_3_–50B_2_O_3_ and 50Y_2_O_3_–50B_2_O_3_ glasses. In contrast, the δ_cs_ value of BO_4_ was greater for the 50La_2_O_3_–50B_2_O_3_ glass than for the 50Y_2_O_3_–50B_2_O_3_ glass. This difference can be partially attributed to the smaller magnetic shielding effect of La^3+^ than of Y^3+^ due to its larger ionic radius. The shift in the *δ*_cs_ values is larger for the BO_4_ peak than for the BO_3_ peak, suggesting that additional structural changes occurred in BO_4_. This may be due to the same higher-parts per million shift of the BO_4_ peak observed in the ^11^B MAS NMR spectra of the La-rich compositions of 50LY. Considering that the *P*_Q_ values are comparable, the BO_4_ units in the La_2_O_3_-rich compositions of 50LY may exhibit a longer B–O bond length than the Y_2_O_3_-rich compositions and not a difference in connectivity.

The BO_3_ units in the 50La_2_O_3_–50B_2_O_3_ glass, which were identified on the basis of Raman scattering spectra and other measurements as isolated BO_3_, exhibit a δ_cs_ value (20.4 ppm) that is smaller than that of *c*-LaBO_3_ (21.2 ppm), which has the same composition ratio and a similar local structure. Upon comparison of the spectral shapes, the peaks in the glass sample are broadened toward lower parts per million. In previous research on alkali borosilicate glasses^41^ and aluminoborosilicate^42^ glasses, it was observed that when there are multiple types of bonds, peak overlap is observed in the 3QMAS NMR spectra. They labeled this as lower parts per million for ring units and higher parts per million for nonring units. However, the overlapping of peaks corresponding to ring or nonring BO_3_ was not observed in this spectrum, and the *P*_Q_ values of the 50La_2_O_3_–50B_2_O_3_ and 50Y_2_O_3_–50B_2_O_3_ glasses are comparable to those of *c*-LaBO_3_. This suggests that BO_3_ has a single local structure without multiple types of bonds. Thus, the BO_3_ units in the glass should form an isolated BO_3_. Therefore, the difference between the glass and crystals can be attributed to the broadening of the distribution of the bond length and distortion of the local structure characteristic to amorphous structures. The latter should contribute to an increase in the nuclear quadrupolar interaction, because of the decrease in the symmetry of the BO_3_ units. However, the *P*_Q_ of the glasses did not show a considerable change compared to that of *c*-LaBO_3_. Hence, no clear structural distortion was observed in the glass; i.e., three-dimensional distortion due to changes in the bond angle or asymmetric two-dimensional distortions of the triangular-planar BO_3_ units did not occur in the glasses. The broadening of the BO_3_ peak occurred only in the lower-parts per million direction. The short-range spread of the bond length distribution is the main factor causing this lower-parts per million broadening while maintaining a symmetry similar to that of a crystal. Considering that the density of the 50La_2_O_3_–50B_2_O_3_ glass [5.54 g/cm^3^ (see Table 1)] is greater than that of *c*-LaBO_3_ (5.25 g/cm^3^),^27^ the average bond length of glass is assumed to be shorter than that of crystals.

The spectral shapes of the peaks and the NMR parameters of the BO_4_ units in the *c*-YBO_3_ and 50Y_2_O_3_–50B_2_O_3_ glass are different (Figure 4b,d and Table 2). In the glass, the peak is along the chemical shift axis (slope = 1), whereas in the crystal, it is parallel to the MAS axis. *P*_Q_ reflects the difference in the spectral shape between the glass and the crystal and is smaller in the glass (0.77 MHz) than in the crystal (0.96 MHz). The nuclear quadrupolar interaction is weaker in the glass. As observed in ^11^B MAS NMR, it shifted to higher parts per million in the glass. Therefore, the BO_4_ units in the glass would be different from those in a crystal with the same composition.

Finally, the possible form of the BO_4_ units in the highly modified borate glasses is discussed. *O*_nb_ in the BO_4_ units can vary from 0 to 4. The BØ_2_O_2_^3–^ in the B_3_O_9_ ring, which is the main structural unit of *c*-YBO_3_, has two *O*_nb_ groups (*O*_nb_ = 2). The BØ_2_O_2_^3–^ unit is considered the lowest-symmetry unit because it has the largest spatial distribution of negative charges. When the value of *O*_nb_ changed from 2, the symmetry of BO_4_ improved and *P*_Q_ decreased. In particular, with an increase in the level of *O*_nb_, the magnetic shielding effect decreased, causing a chemical shift of the BO_4_ unit toward the higher-parts per million side. Therefore, the difference observed in the spectra of the 50Y_2_O_3_–50B_2_O_3_ glass and *c*-YBO_3_ was attributed to an increase in *O*_nb_. Thus, the BO_4_ units in the 50Y_2_O_3_–50B_2_O_3_ glass should have an *O*_nb_ of >2. Therefore, the candidates for the BO_4_ unit in the glass are narrowed down to isolated BO_4_ (*O*_nb_ = 4), the B_2_O_7_ dimer (*O*_nb_ = 3), and the B_3_O_10_ trimer (averaged *O*_nb_ = 2.67), which is generated through B_3_O_9_ ring opening. Among them, the B_3_O_10_ trimer has two types of BO_4_: one in the center of the trimer with an *O*_nb_ of 2 and the other with an *O*_nb_ of 3. The B_3_O_10_ trimer is a large chain unit compared to the isolated BO_4_ and B_2_O_7_ dimer. Considering that BO_3_, which is the main structural unit in the glass, exists only as isolated BO_3_ units, it is not reasonable to assume that BO_4_ selectively aggregates to form longer chain units. Considering that the ionic radii of La^3+^ and Y^3+^ are close to that of O^2–^ and much larger than that of B^3+^, it is assumed that the space is filled with La^3+^, Y^3+^, and O^2–^ in La_2_O_3_ and Y_2_O_3_-rich borate glasses and that B^3+^ is inserted into the interstitial sites generated by three or four O^2–^ ions. Around Y^3+^, which has an ionic radius that is smaller than that of La^3+^ and is more ionically bonded Y–O, B atoms are more easily incorporated into interstitial sites, resulting in the formation of more BO_4_. Thus, these BO_4_ units connect locally to produce small structural units. In addition to the value of *O*_nb_, the difference in the B–O bond length should be considered as a factor affecting the NMR spectra; however, it is difficult to experimentally separate the effect of the B–O bond length from that of the increase in *O*_nb_. Nevertheless, the possible forms of BO_4_ in the highly modified glass are determined to be small structural units such as isolated BO_4_, the B_2_O_7_ dimer, and the B_3_O_10_ trimer.

### Dependence of B–O Coordination Number on Composition

**Figure 6:**
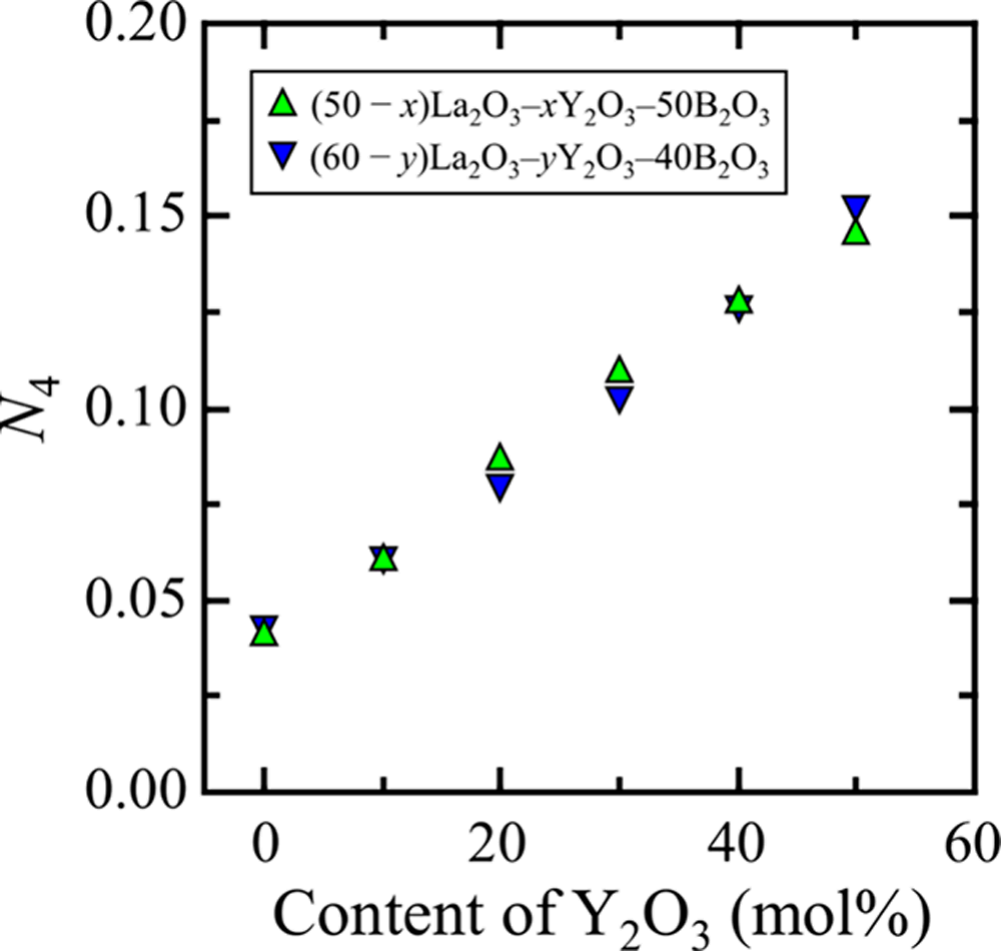
Composition dependence of the *N*_4_ values of La_2_O_3_–Y_2_O_3_–B_2_O_3_ ternary glasses.

The fractions of the BO_3_ and BO_4_ units were estimated using the integral of the fitted curves and used to calculate the *N*_4_ value (Figure 6 and Table 1). *N*_4_ gradually increased with Y_2_O_3_ content. Previous studies concluded that 50La_2_O_3_–50B_2_O_3_ and 60La_2_O_3_–40B_2_O_3_ do not contain BO_4_. However, the careful fitting conducted in this study revealed that ∼4% of BO_4_ survives in the 50La_2_O_3_–50B_2_O_3_ glass. A recent report using high-field (19.6 T) ^11^B MAS NMR supports the presence of BO_4_.^36^ The data of 60LY ternary glasses and Y_2_O_3_–B_2_O_3_ binary glasses were also fitted, and the *N*_4_ values were estimated (Figures S6 and S7 and Tables S1 and S2).

**Figure 7:**
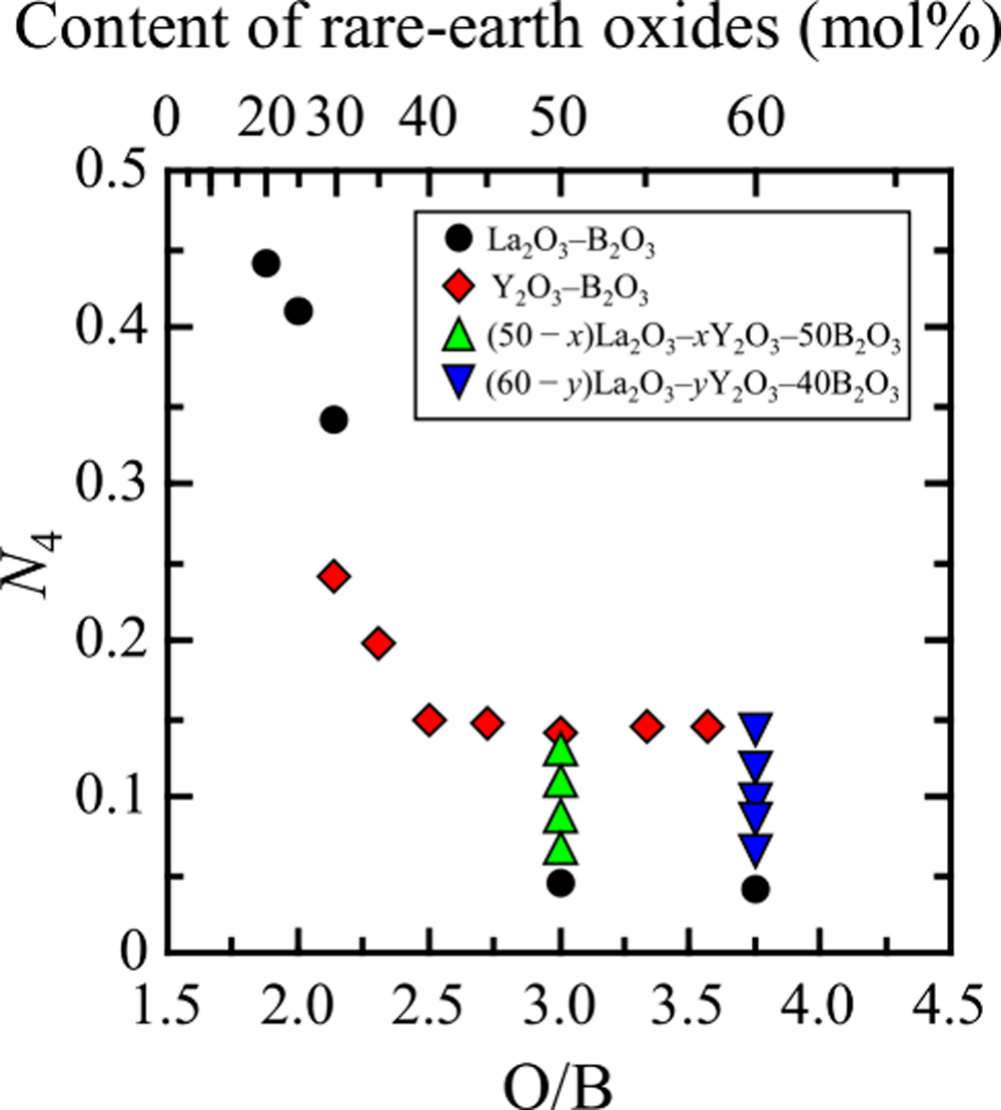
*N*_4_ values of the La_2_O_3_–B_2_O_3_ glasses (black circles), Y_2_O_3_–B_2_O_3_ glasses (red squares), and La_2_O_3_–Y_2_O_3_–B_2_O_3_ (triangles) as a function of the O/B ratio. The data of the La_2_O_3_–B_2_O_3_ glass with a low La_2_O_3_ content (O/B < 2.2) were obtained from ref (21).

The *N*_4_ values estimated from the fitting are plotted versus the O/B ratio, which reflects the glass compositions (Figure 7). When O/B ≤ 2.5, the *N*_4_ changes are in good agreement with a previous study on conventional alkali and alkaline-earth borate glasses.^7,8,21^ In this composition range, the types of modifier oxides exhibited no strong effect on the *N*_4_ values. When O/B ≥ 3, the oxygen atoms introduced by the modifier oxide were sufficient to break all of the B–O–B bonds and form isolated BO_3_. Therefore, the *N*_4_ value should be zero. However, the *N*_4_ values are finite in the rare-earth-rich borate glasses. This means that the introduction of O from rare-earth oxides does not contribute to the formation of BO_3_. The *N*_4_ values were ∼0.04 and ∼0.15 in the La_2_O_3_–B_2_O_3_ and Y_2_O_3_–B_2_O_3_ glasses, respectively. Furthermore, the *N*_4_ values at O/B ≥ 3 exhibited only slight changes with an increase in the O/B ratio, even though different La_2_O_3_ and Y_2_O_3_ contents resulted in different *N*_4_ values. The *N*_4_ value increased with Y_2_O_3_ content. These results suggest that Y^3+^ is more likely to form BO_4_ than La^3+^. Therefore, it is necessary to focus on the difference between the effects of La^3+^ and Y^3+^ on the formation of BO_4_.

### *Ab Initio* Calculation of Crystals

To compare the effects of La^3+^ and Y^3+^ on the connectivity of the BO_*n*_ units, electronic structure analysis of the structures of *c*-LaBO_3_ and *c*-YBO_3_ optimized by *ab initio* calculations was performed.^44^ After ionic relaxation, the lattice constants (*a*, *b*, and *c*) of *c*-LaBO_3_ were 5.044, 8.179, and 5.628, respectively, and those of *c*-YBO_3_ were 11.146, 6.429, and 8.761, respectively. These values are in good agreement with the experimental values within 5%.

Grid-based Bader charges were computed by partitioning real space based on neighboring atoms and integrating the electron density within the assigned space of each atom. In *c*-LaBO_3_, the partial charge of B in the isolated BO_3_ unit was 2.22 e. In *c*-YBO_3_, the partial charge of B in the B_3_O_9_ unit was 2.27–2.29 e. There was no considerable difference between the partial charges of the three-coordinated and four-coordinated B. This was an unexpected result because the charge localization for B is the same for three-coordinated B and four-coordinated B. Thus, the difference between La^3+^ and Y^3+^ should not have a strong effect on the partial charge of boron.

**Figure 8:**
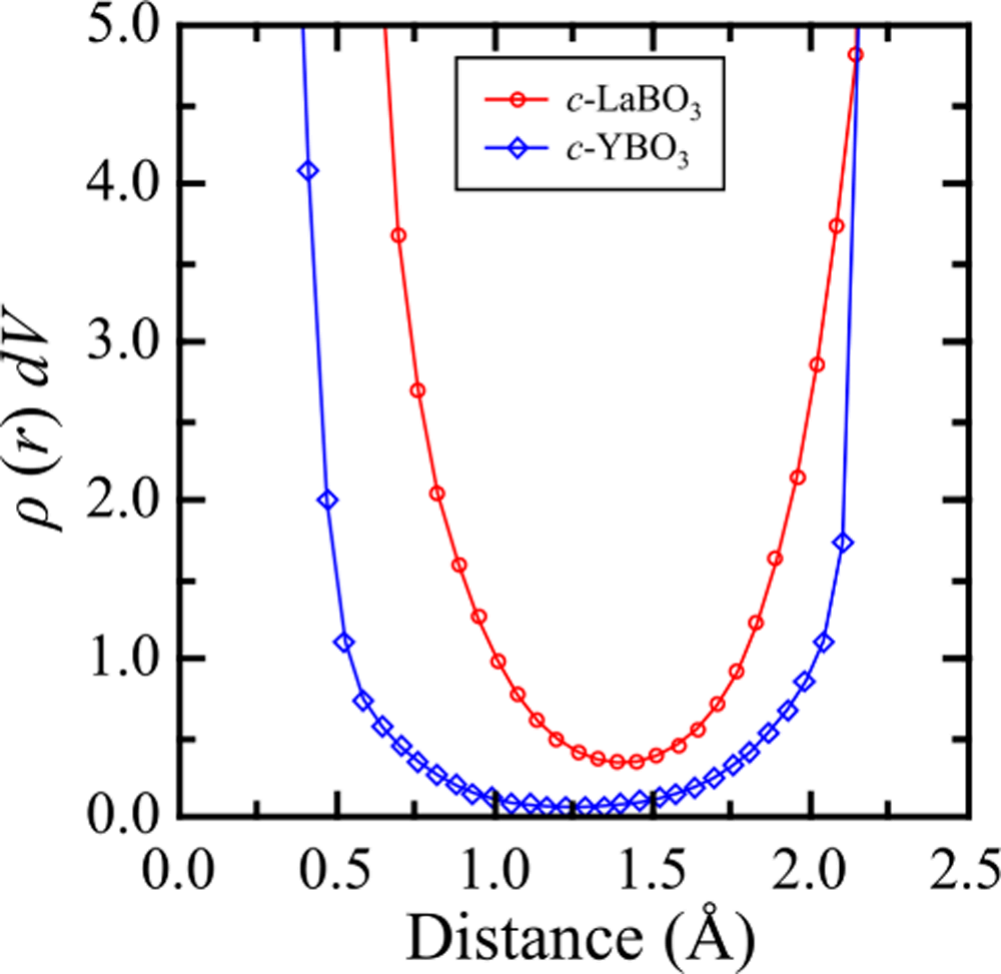
Spatial line analysis of the electron density within the La–O bonds and Y–O bonds of *c*-LaBO_3_ and *c*-YBO_3_, respectively. The electron density at each point is calculated through the interpolation of the electron density from the surrounding voxel.

The electron density profile at points on the line connecting R and O (R = La or Y) was calculated on the basis of electron density distribution. The electron density for 40 points from a segmented line was obtained by interpolation around the voxels. Figure 8 illustrates the mean spatial distribution of electrons multiplied by voxel volume *dV* between the R–O bonds. In *c*-LaBO_3_, the electron density of the La–O bond pairs exhibited a narrow distribution with a minimum position around *r* = 1.39 Å. In contrast, there is a wide region in which the electron density of the Y–O bond pairs is close to zero. This indicates that the electrons in the Y–O bond tend to localize near the atomic nuclei compared to those in the La–O bonds in crystals at the same B_2_O_3_ ratio. This suggests that the ionically bonded R–O correlates with the formation of BO_4_ units with large negatively charged O atoms. This is also expected to occur in the case of bonding of Y^3+^ and La^3+^ in glasses. The increase in the level of Y^3+^ at the same B_2_O_3_ ratio causes the localization of electrons near the atomic nuclei. Consequently, higher Y_2_O_3_ contents can increase the production of the BO_4_ units, which contribute to the increase in *N*_4_. This indicates that local structures in highly modified borate glasses may be controlled by designing compositions on the basis of the understanding of the nature of the chemical bonds between modifier cations and oxygen.

## Conclusions

La_2_O_3_–Y_2_O_3_–B_2_O_3_ ternary glass was synthesized by using an ADL furnace. The local structure around the B atoms was investigated using several spectroscopy analyses. In the 50LY glasses, Raman scattering spectra indicated that all BO_3_ units were isolated, and BO_4_ peaks were not observed. The ^11^B 3QMAS NMR spectra proved the presence of BO_4_ units in the 50LY glasses. *N*_4_ increased with the Y_2_O_3_ content in the glass. Comparison of the ^11^B 3QMAS NMR spectra of the 50La_2_O_3_–50B_2_O_3_ glass and the LaBO_3_ crystal suggests that the B–O bond lengths of isolated BO_3_ in the glass are shorter, and their distribution is broader than those in the crystal. The number of nonbridging oxygens of BO_4_ in the 50Y_2_O_3_–50B_2_O_3_ glass was estimated by comparing its NMR parameters with those of the YBO_3_ crystal to be >2. Thus, its structure was considered to be fragmented, similar to that of the isolated BO_4_ unit. Thus, La_2_O_3_- and Y_2_O_3_-rich borate glass have a characteristic fragmented structure with no three-dimensional random network. This was clarified using *ab initio* calculation analysis of the crystals to be the result of the electrons in the Y–O bond tending to localize near the atomic nuclei than those in the La–O bonds. Therefore, Y^3+^ is more likely to form BO_4_ than La^3+^, and the selective addition of these cations to borate glass can be used to control its structural units.^43^

## Supporting Information Available

Raman scattering spectra of (60 – y)La_2_O_3_*–y*Y_2_O_3_–40B_2_O_3_ glasses and *z*Y_2_O_3_–(100 – z)B_2_O_3_ glasses, ^11^B MAS NMR spectra of (50 – *x*)La_2_O_3_–*x*Y_2_O_3_–50B_2_O_3_ ternary glasses, (60 – *y*)La_2_O_3_–*y*Y_2_O_3_–40B_2_O_3_ ternary glasses, and *z*Y_2_O_3_–(100 – *z*)B_2_O_3_ binary glasses and NMR parameters of (60 – *y*)La_2_O_3_–*y*Y_2_O_3_–40B_2_O_3_ ternary glasses and *z*Y_2_O_3_–(100 – *z*)B_2_O_3_ binary glasses (PDF)

## Abbreviations

^11^B MAS NMR: ^11^B magic angle spinning nuclear magnetic resonance
XRD: X-ray diffraction
ADL: aerodynamic levitation
DFT: density functional theory
GGA–PBE: generalized gradient approximation with Perdew–Burke–Ernzerhof
QIS: quadrupolar induced shift
