# Supplementary material for: Survival of Fragmented BO4 Units in Highly Modified Rare-Earth-Rich Borate Glasses
Source: Inorg Chem. 2024 Nov 20;63(49):23131–40. doi: 10.1021/acs.inorgchem.4c03264 (PMC11632762; doi:10.1021/acs.inorgchem.4c03264)
Supplement: Supplementary file 1 — ic4c03264_si_001.pdf [file ic4c03264_si_001.pdf]

Supplemental Information:
Survival of fragmentated BO_4 units in highly modified rare-earth-rich borate glasses

Shunta Sasaki,[1] Atsunobu Masuno,[1,2,3*] Yutaka Yanaba,[3] Hiroyuki Inoue[3], Takahiro Ohkubo[4]

[1]Graduate School of Science and Technology, Hirosaki University, 3 Bunkyo-cho, Hirosaki, Aomori 036-8505, Japan

[2] Graduate School of Engineering, Kyoto University, Kyotodaigaku-Katsura, Nishikyo-ku, Kyoto 615-8520, Japan

[3]Institute of Industrial Science, The University of Tokyo, 4-6-1 Komaba, Meguro-ku, Tokyo 153-8505, Japan

[4]Graduate School of Engineering, Chiba University, 1-33 Yayoi-cho Inage-ku, Chiba 263-8522, Japan

*Corresponding author. E-mail: masuno.atsunobu.3k@kyoto-u.ac.jp

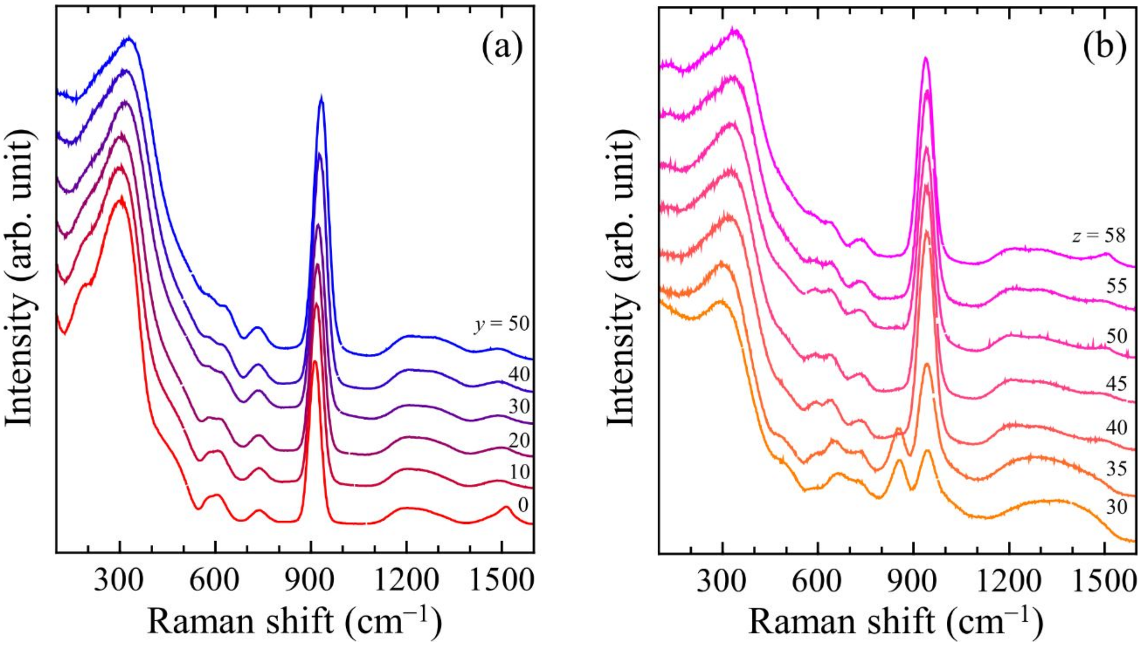

Figure S1. Raman scattering spectra of (a) (60 − *y*)La_2O_3–*y*Y_2O_3–40B_2O_3 glasses and (b) *z*Y_2O_3–(100 − *z*)B_2O_3 glasses.

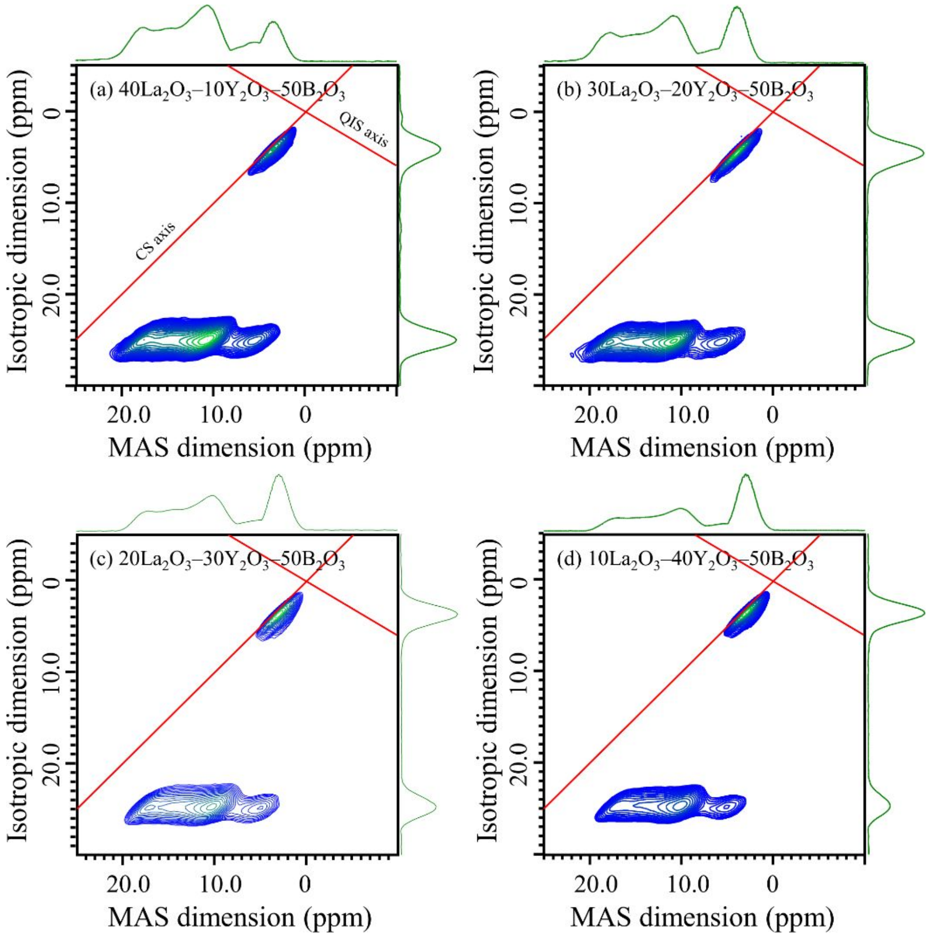

Figure S2. ^{11}B 3QMAS NMR spectra of (a) $40La_2O_3$–$10Y_2O_3$–$50B_2O_3$, (b) $30La_2O_3$–$20Y_2O_3$–$50B_2O_3$, (c) $20La_2O_3$–$30Y_2O_3$–$50B_2O_3$ and (d) $10La_2O_3$–$40Y_2O_3$–$50B_2O_3$ glasses. A chemical shift (CS) axis with a slope of 1 and a quadrupolar induced shift (QIS) axis with a slope of −10/17 are inserted.

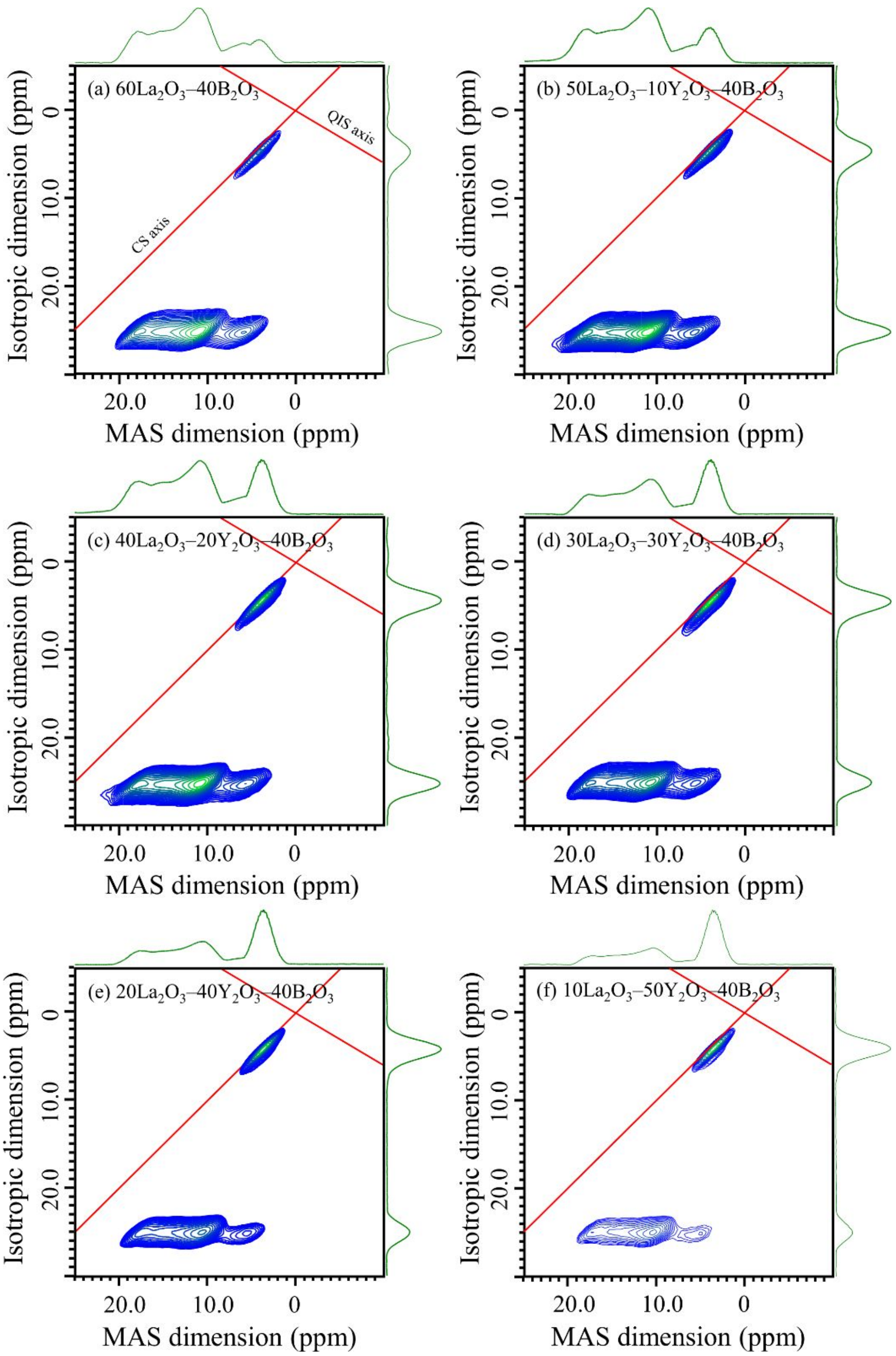

Figure S3. ^{11}B 3QMAS NMR spectra of (a) $60La_2O_3$–$40B_2O_3$, (b) $50La_2O_3$–$10Y_2O_3$–$40B_2O_3$, (c) $40La_2O_3$–$20Y_2O_3$–$40B_2O_3$, (d) $30La_2O_3$–$30Y_2O_3$–$40B_2O_3$, (e) $20La_2O_3$–$40Y_2O_3$–$40B_2O_3$, and (f) $10La_2O_3$–$50Y_2O_3$–$40B_2O_3$ glasses. A chemical shift (CS) axis with a slope of 1 and a quadrupolar induced shift (QIS) axis with a slope of −10/17 are inserted.

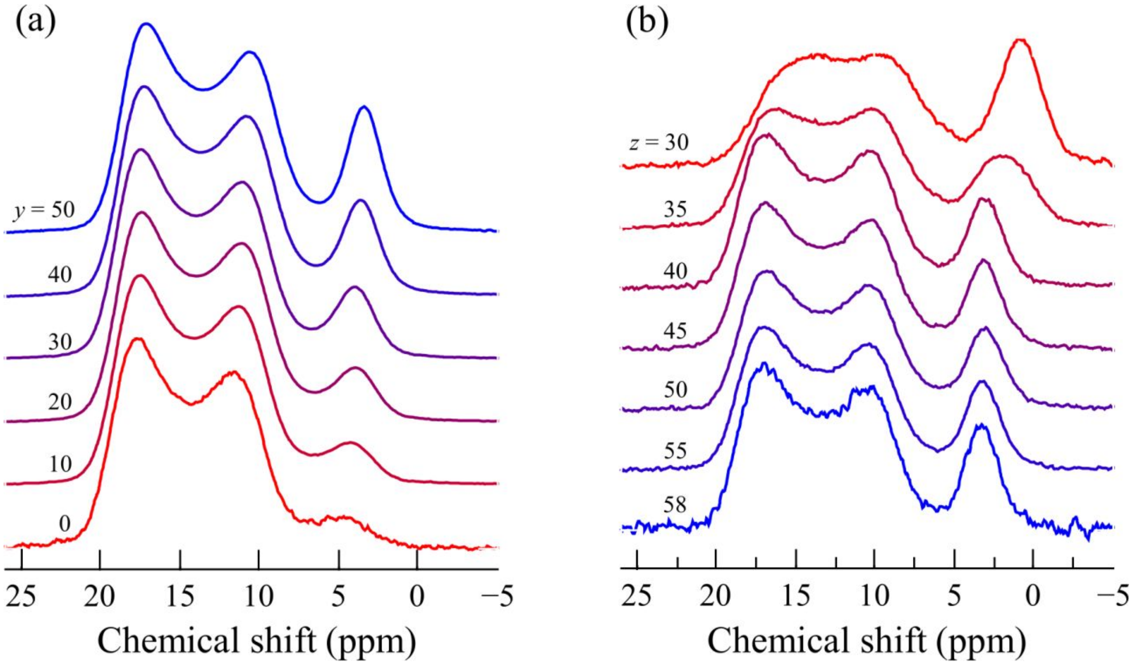

Figure S4. ^{11}B MAS NMR spectra of (a) $(60 - y)La_2O_3$–yY_2O_3–$40B_2O_3$ glasses and (b) zY_2O_3–$(100 - z)B_2O_3$ glasses.

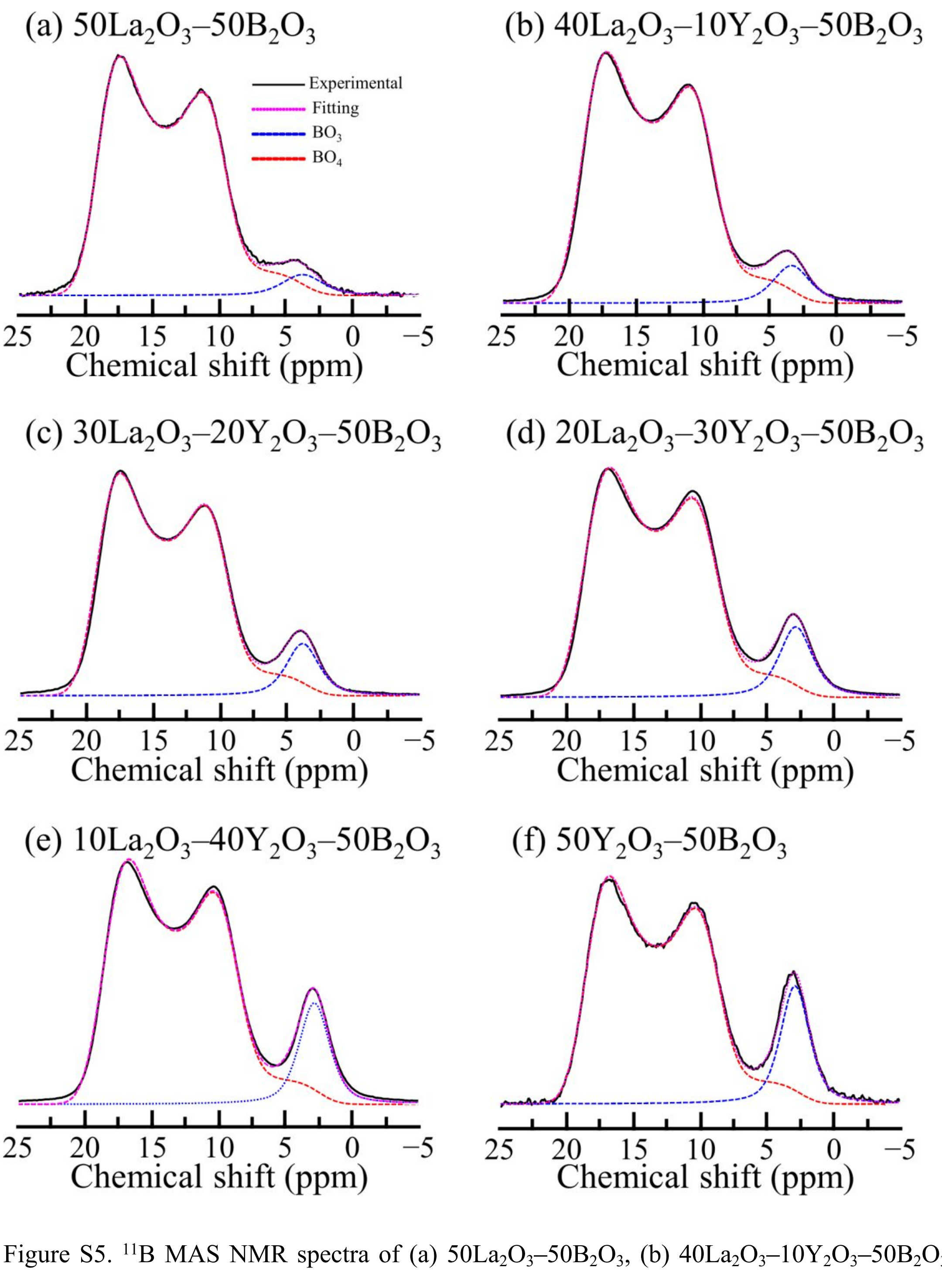

Figure S5. ^{11}B MAS NMR spectra of (a) $50La_2O_3–50B_2O_3$, (b) $40La_2O_3–10Y_2O_3–50B_2O_3$, (c) $30La_2O_3–20Y_2O_3–50B_2O_3$, (d) $20La_2O_3–30Y_2O_3–50B_2O_3$, (e) $10La_2O_3–40Y_2O_3–50B_2O_3$, and (f) $50Y_2O_3–50B_2O_3$ glasses. Solid lines represent experimental data, dotted lines represent fitting data, and dash lines represent each component of the fitting.

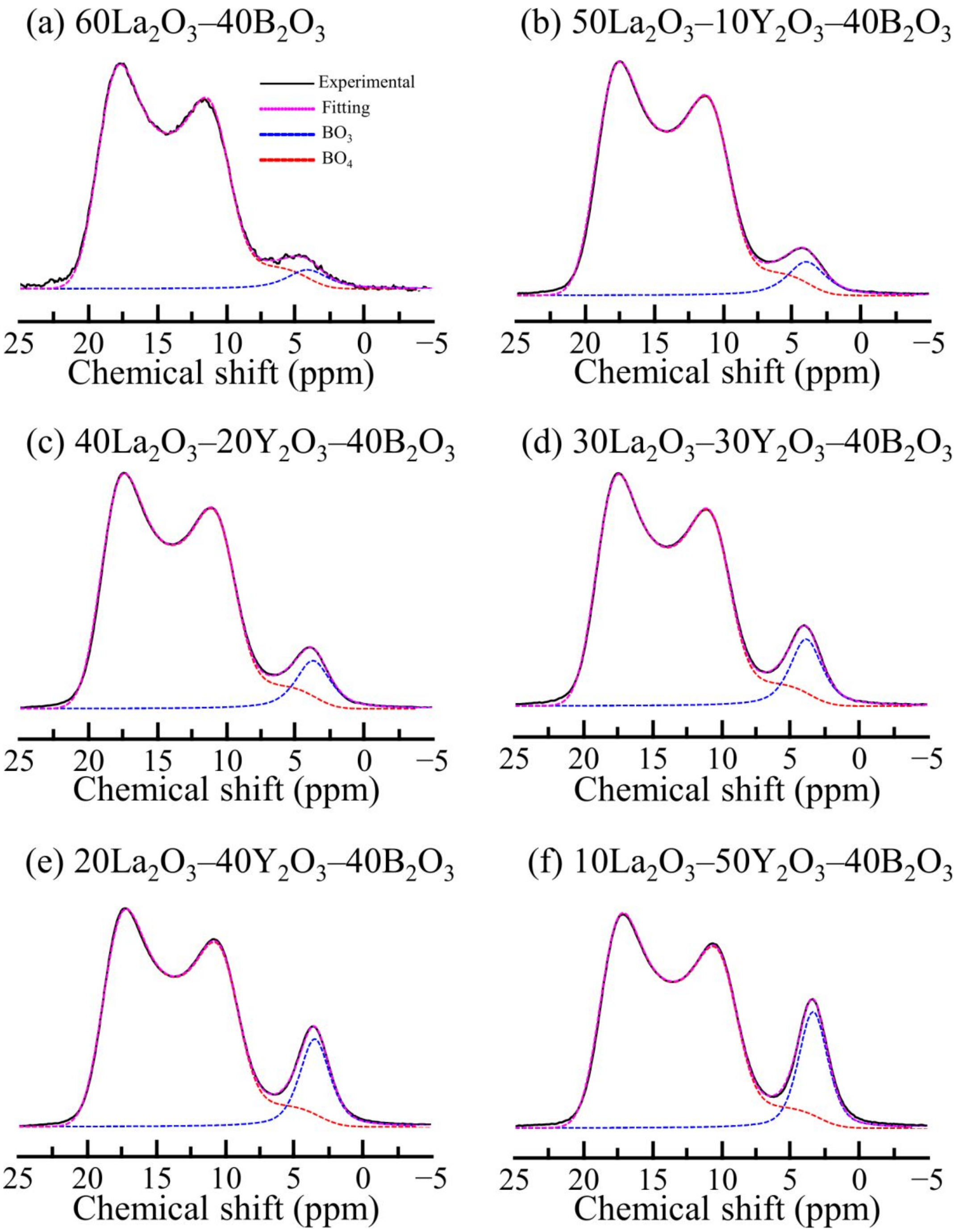

Figure S6. ^{11}B MAS NMR spectra of (a) $60La_2O_3–40B_2O_3$, (b) $50La_2O_3–10Y_2O_3–40B_2O_3$, (c) $40La_2O_3–20Y_2O_3–40B_2O_3$, (d) $30La_2O_3–30Y_2O_3–40B_2O_3$, (e) $20La_2O_3–40Y_2O_3–40B_2O_3$, and (f) $10La_2O_3–50Y_2O_3–40B_2O_3$ glasses. Solid lines represent experimental data, dotted lines represent fitting data, and dash lines represent each component of the fitting.

Table S1. ^{11}B MAS NMR parameters: isotropic chemical shift (δ_{iso}), quadrupole coupling constant (C_Q), quadrupole asymmetry parameter (η) and Gaussian broadening of $(60 - y)La_2O_3$–yY_2O_3–$40B_2O_3$ ternary glasses extracted from spectral decompositions using the Gaussian/Lorentzian and Q MAS 1/2 models for the BO_4 and BO_3 peaks, respectively. ^{11}B 3QMAS NMR parameters: chemical shift (δ_{cs}) and magnitude of the nuclear quadrupolar interaction (P_Q) of the BO_4 and BO_3 peaks.

	δ_{iso} (ppm)	C_Q (MHz)	η	Gaussian broadning (Hz)	δ_{cs} (ppm)	P_Q (MHz)
$60La_2O_3$–$40B_2O_3$						
BO_4	4.1	—	—	—	4.8	0.73
BO_3	21.2	2.66	0.10	441.3	20.4	2.87
$50La_2O_3$–$10Y_2O$–$40B_2O_3$						
BO_4	4.0	—	—	—	4.5	0.71
BO_3	21.1	2.68	0.14	435.2	20.5	2.85
$40La_2O_3$–$20Y_2O$–$40B_2O_3$						
BO_4	3.7	—	—	—	4.3	0.69
BO_3	21.0	2.69	0.12	443.8	20.7	2.87
$30La_2O_3$–$30Y_2O$–$40B_2O_3$						
BO_4	3.9	—	—	—	4.5	0.75
BO_3	21.0	2.69	0.10	438.5	20.3	2.89
$20La_2O_3$–$40Y_2O$–$40B_2O_3$						
BO_4	3.5	—	—	—	5.0	1.22
BO_3	20.8	2.70	0.10	448.1	21.0	3.06
$10La_2O_3$–$50Y_2O$–$40B_2O_3$						
BO_4	3.3	—	—	—	4.0	0.71
BO_3	20.6	2.73	0.20	416.8	20.1	2.92

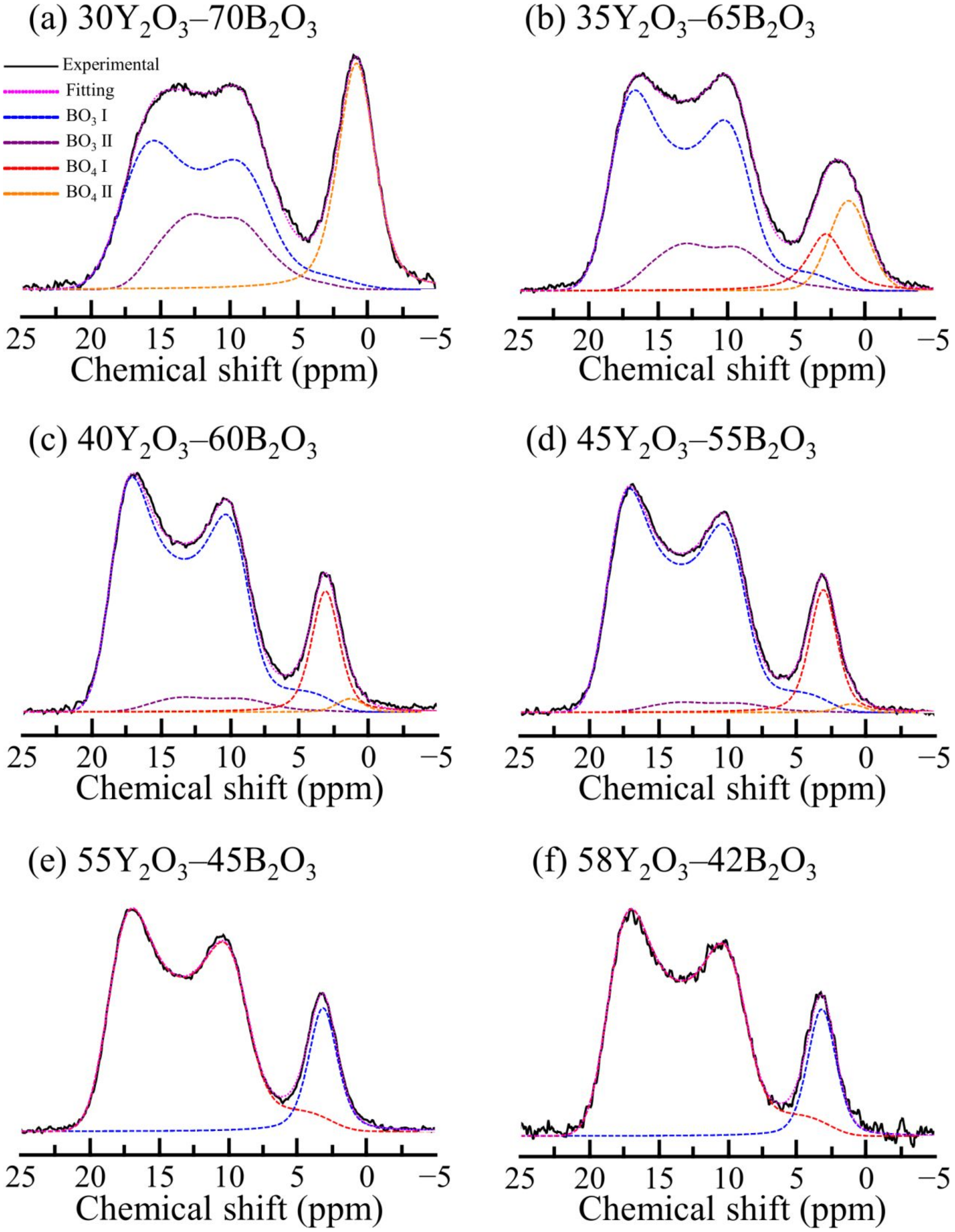

Figure S7 ^{11}B MAS NMR spectra of zY_2O_3–$(100 - z)B_2O_3$ binary glasses. Solid lines represent experimental data, dotted lines represent fitting data, and dash lines represent each component of the fitting.

Table S2. ^{11}B NMR parameters of zY_2O_3–$(100 - z)B_2O_3$ binary glasses. The peaks for BO_3 and BO_4 were labeled in order from the low magnetic field side as I and II. N_4 values for each composition are shown together.

	δ_{iso} (ppm)	Fraction (%)		δ_{iso} (ppm)	C_Q (MHz)	η	Gaussian broadning (Hz)	Fraction (%)	N_4
$58Y_2O_3$–$42B_2O_3$									0.15
BO_4 I	3.2	14.8	BO_3 I	20.6	2.73	0.10	451.5	85.2	
BO_4 II	–	0	BO_3 II	—	—	—	—	0	
$55Y_2O$–$45B_2O_3$									0.15
BO_4 I	3.2	15.1	BO_3 I	20.6	2.73	0.10	465.0	84.9	
BO_4 II	–	0	BO_3 II	—	—	—	—	0	
$45Y_2O$–$55B_2O_3$									0.15
BO_4 I	3.2	13.5	BO_3 I	20.6	2.72	0.09	464.7	85.4	
BO_4 II	1.1	1.1	BO_3 II	—	—	—		0	
$40Y_2O$–$60B_2O_3$									0.14
BO_4 I	3.1	13.6	BO_3 I	20.7	2.73	0.09	418.9	80.0	
BO_4 II	1.3	1.3	BO_3 II	17.4	2.47	0.35	476.5	5.1	
$35Y_2O$–$65B_2O_3$									0.18
BO_4 I	2.8	8.5	BO_3 I	20.4	2.74	0.07	496.2	65.9	
BO_4 II	1.2	11.2	BO_3 II	17.2	2.45	0.40	420.0	14.5	
$30Y_2O_3$–$70B_2O_3$									0.25
BO_4 I	–	0	BO_3 I	19.6	2.76	0.08	628.3	50.6	
BO_4 II	0.8	28.4	BO_3 II	16.9	2.41	0.43	434.1	21.1	
